# Ingesting yeast extract causes excitation of neurogenic and myogenic colonic motor patterns in the rat

**DOI:** 10.1111/jcmm.18343

**Published:** 2024-05-17

**Authors:** Hongfei Li, Yanzhao Ji, Hesheng Luo, Jan D. Huizinga, Ji‐Hong Chen

**Affiliations:** ^1^ Shanxi Medical University Taiyuan Shanxi China; ^2^ Shanxi Bethune Hospital, Shanxi Academy of Medical Sciences, Tongji Shanxi Hospital, Third Hospital of Shanxi Medical University Taiyuan Shanxi China; ^3^ Department of Gastroenterology and Hepatology Renmin Hospital of Wuhan University, Key Laboratory of Hubei Province for Digestive System Diseases Wuhan Hubei China; ^4^ Department of Medicine Farncombe Family Digestive Health Research Institute, McMaster University Hamilton Ontario Canada

**Keywords:** autonomic nervous system, colonic motility, myogenic activity, spatiotemporal mapping, yeast extract

## Abstract

Fermented foods play a significant role in the human diet for their natural, highly nutritious and healthy attributes. Our aim was to study the effect of yeast extract, a fermented substance extracted from natural yeast, on colonic motility to better understand its potential therapeutic role. A yeast extract was given to rats by gavage for 3 days, and myogenic and neurogenic components of colonic motility were studied using spatiotemporal maps made from video recordings of the whole colon ex vivo. A control group received saline gavages. The yeast extract caused excitation of the musculature by increasing the propagation length and duration of long‐distance contractions, the major propulsive activity of the rat colon. The yeast extract also evoked rhythmic propulsive motor complexes (RPMCs) which were antegrade in the proximal and mid‐colon and retrograde in the distal colon. RPMC activity was evoked by distention‐induced neural activity, but it was myogenic in nature since we showed it to be generated by bethanechol in the presence of tetrodotoxin. In conclusion, ingestion of yeast extract stimulates rat colon motility by exciting neurogenic and myogenic control mechanisms.

## 1 INTRODUCTION

Fermented foods play an important role in our diet to induce specific health benefits through effects on the microbiota,^1^, ^2^, ^3^ which helps digest complex dietary substrates,^4^ provides synthesis of important nutrients,^5^ prevents pathogenic bacteria colonization, activates the immune system,^6^ and stimulates hormonal pathways which affect many aspects of host physiology.^7^ Fermented foods have a long history of at least 10,000 years. The use of probiotics historically focused on gastrointestinal disease, including infectious gastroenteritis, functional disturbances and immune‐inflammatory disorders, for example, the use of fermented milk to changes the gut microbiome.^8^ Fermented foods make up almost 1/3 of our diet^9^; their metabolites, such as acids, carbon dioxide and vitamins, aid in health, notably short‐chain fatty acids (SCFAs), which are undigested until reaching the colon.^10^ SCFAs include acetate, propionate and butyrate,^11^ providing multiple benefits for the host as an energy source and triggering hormone pathways.^12^ For instance, butyrate has an effect in accelerating cholinergic maturation and enhancing cholinergic expression in ageing neurons, which could be of therapeutic interest in gastrointestinal disorders associated with inhibition of colonic transit.^13^

Yeasts are good candidates as probiotics because yeasts entering the gastrointestinal tract are resistant to local stresses such as the presence of enzymes, bile salts, organic acids and considerable variations of pH and temperature, including high acidity.^14^

Yeasts are single‐cell micro‐organisms belonging to the fungus family and can quickly multiply by means of cell division. Yeast extract is obtained from natural yeast through autolyzing baker's yeast or brewers' yeast,^15^ degrading the proteins and nucleic acids from the cell wall, making valuable components from the yeast cells available to the human body. It contains multiple nutritional ingredients: more than 20 amino acids, B vitamins, microelements such as calcium and potassium and minerals such as zinc and iron. Yeast extract has been widely used as a food additive or flavouring or as nutrients for bacterial culture media for the past 50 years.^14^ EpiCor® fermentate is a nutrient rich, high‐metabolite yeast product of fermented *Saccharomyces cerevisiae* (*S. cerevisiae*); it has significant antioxidant activity, and potent immune‐modulatory and anti‐inflammatory effects in healthy adults.^16^, ^17^, ^18^ Furthermore, EpiCor fermentate was reported to improve constipation‐associated symptoms by changing the composition of the gut microbiome.^19^ Another study suggested that the probiotic strain *S. cerevisiae* may be useful in the fight against *Enterohemorrhagic Escherichia coli* infection by upregulating the stx and eae genes (two main virulence factors). Furthermore, the pathogen showed a donor‐dependent effect on gut microbiota and may be antagonized by probiotic yeast during interaction with Peyer's patches.^20^ Studies using bread yeast cell wall β‐glucans on mice showed enhanced intestinal motility and regulation of the expression of neurotransmitters and tight junction protein.^21^ Triple‐fermented barley extracts act as a promising laxative agent and functional food ingredient to reduce spastic constipation.^22^ Furthermore, a recent study demonstrated that yeast extract had rapid itch relief in chronic pruritus as it blocked various histamine receptors and inhibited numerous inflammatory cytokines.^23^

**FIGURE 1:**
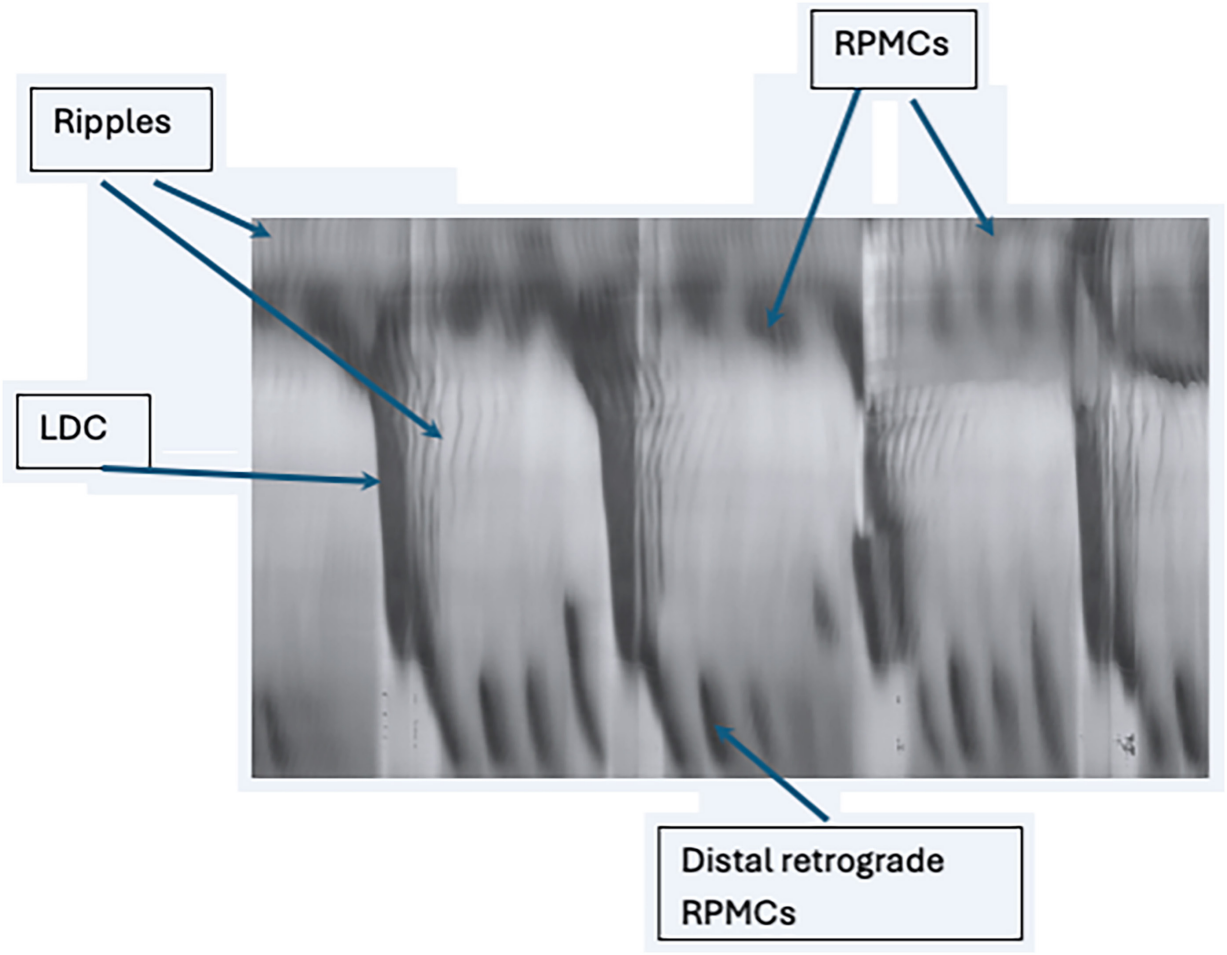
The motor patterns of the rat colon.

Our objective was to study the effect of yeast extract on gastrointestinal motility, focusing on its effects on the motor patterns of the rat colon^24^ (Figure 1). The major pan‐colonic motor patterns include long‐distance contractions (LDC)^24^; LDCs originate from the proximal colon and propagate most often to the distal end, covering at least 2/3 of the colon, moving its content in anal direction. They are the equivalent of the High amplitude propagating pressure waves (HAPWs) in the human colon.^25^ LDCs consist of two components, a contraction phase lasting about 30 s, preceded by a relaxation phase lasting about 10 s.

Rhythmic propulsive motor complexes (RPMCs) start from the proximal, mid or distal colon, propagating in anal direction with amplitudes as high as that of LDCs. RPMCs have a shorter propagation length and a slower propagation velocity; when antegrade, they are effective in clearing the colon. RPMCs are propagating ring contractions without the sustained component of the LDC and most often do not have a relaxation phase. Distal retrograde contractions are also prominent in the rat colon; their function may be similar to the distal 3 cpm cyclic motor pattern in the human colon.^26^, ^27^, ^28^

Segmentation consists of several (usually 2–5) short‐lasting non‐propulsive rings of contraction occurring at the same time dividing the colon into segments, because of a waxing and waning of the amplitude of the slow wave resulting in a rhythmic chequered pattern of segmentation motor activity, which is a major pattern in the small intestine facilitating the absorption of nutrients.^29^

Ripples originate most often in the proximal colon and are of low amplitude and high frequency; they often change their direction of propagation, governed by slow waves that are generated by the interstitial cells of Cajal of the submuscular plexus (ICC‐SMP).^30^, ^31^

## 2 MATERIALS AND METHODS

### 2.1 Animals

Adult male Sprague–Dawley rats weighing 200–300 g were used (*n* = 28). The study protocol was approved by the Ethics Committee of Renmin Hospital of Wuhan University School of Medicine (NSFC‐81170249).

### 2.2 Preparation

The rats were separated into two groups: an experimental group (n = 18) and a control group (*n* = 10). All the rats in the experimental group were given intragastric yeast extract (0.1 mg dissolved in 10 mL distilled water) administration for 3 days by gavage in the morning. After rats were killed by cervical dislocation, the entire colon was removed and placed in gassed Krebs (5% CO_2_ and 95% O_2_) at 37°C, pH 7.3–~–7.4. Krebs solution consisted of (mM) NaCl 118.1, KCl 4.8, NaHCO_3_ 25, NaH_2_PO_4_ 1.3, MgCl_2_∙6H_2_O 1.2, Glucose 12.2 and CaCl_2_ 2.5. All procedures, organ bath, rat species were identical to those published previously.^24^, ^32^ In short, the colon contents were gently flushed out using a warmed Krebs solution and external connective tissue was removed because that interferes with spatiotemporal mapping. The proximal and distal ends were cannulated and fixed to the bottom of the organ bath. The proximal inflow tube (inner diameter 3 mm, outer diameter 4 mm) was connected to a 50 mL syringe and placed 15 cm above the level of the colon with phosphate buffered solution (PBS with 10 μM indomethacin, without glucose). PBS consisted of NaCl 137.1 mM, KCl 2.7 mM, Na_2_HPO_4_ 10 mM and KH_2_PO_4_ 2 mM. The distal outflow tube (inner diameter 3 mm, outer diameter 4 mm) was positioned in a narrow upright container filled with PBS. The fluid level in the container determined the intraluminal pressure and the standard intraluminal pressure at the beginning was 5 cm H_2_O. After an LDC was completed, fluid flowed back into the colon. The colon was left to equilibrate for 20–30 min before the experiment started. A video camera was mounted above the preparation, and each experiment was recorded. Data acquisition occurred through a Microsoft camera using Microsoft Lifecam software. Drugs were given via the bath solution since the focus was on the actions on the enteric nervous system (ENS). In the tables, ‘n’ refers to the number of animals used.

### 2.3 Materials

The following materials were used: yeast extract (Product # LP0021B; Thermo Fisher Oxoid, Basingstoke, England) tetrodotoxin (TTX; Baoman Biochemistry Co. Ltd., Shanghai, China), bethanechol (3B Scientific Corporation, Libertyville, Illinois, USA).

Krebs and PBS reagents were purchased from Sinopharm Chemical Reagent Co. Ltd., Shanghai, China.

### 2.4 Spatiotemporal mapping

Data acquisition occurred through a Microsoft camera using Microsoft Lifecam software. Video recordings were analysed by image J aided by plugins written by Dr. Sean Parsons.^24^ A ‘spatiotemporal map’ is an image which represents the motor activity based on the change in colon diameter over time. Diagonal streaks of black represent propagating contractions. Colon width is calculated between pixels along the colon's length (image *y*‐axis) for each video frame (image *x*‐axis). Changes in diameter were quantified after calibrating distance, using dots at the bottom of the organ bath, which were separated by exactly 1 cm. The maps were made of the whole colon except for both ends, which were used to fix the colon onto the inflow and outflow tubes; the length studied was between 13 and 19 cm.

### 2.5 Statistics

We used the student's *t* test (Independent Samples *t* test) and a non‐parametric test when equal variance was not achieved, to do the statistical analysis. Data were expressed as mean ± SD.

## 3 RESULTS

### 3.1 Effect of yeast extract on LDCs

**FIGURE 2:**
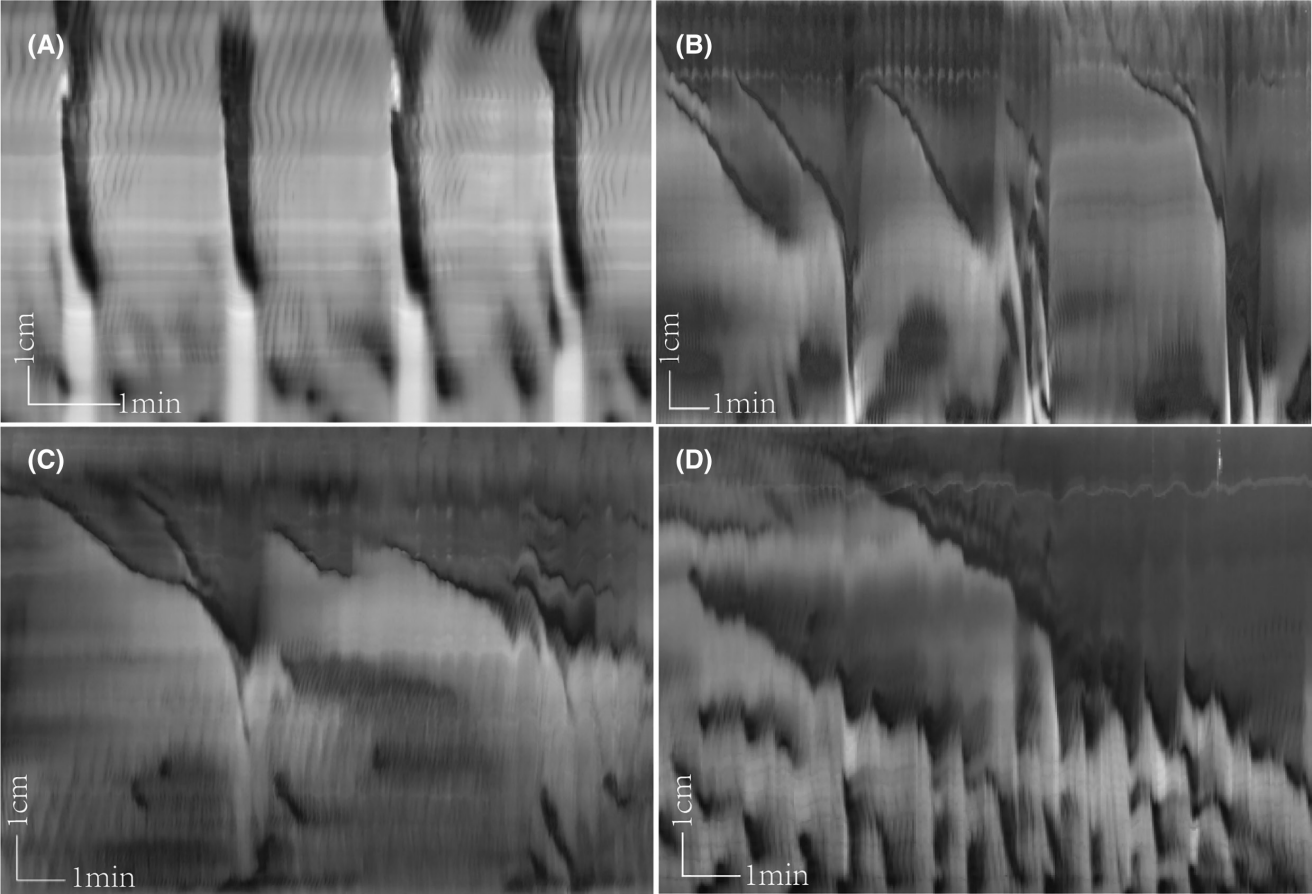
Long‐distance contraction (LDC) activity in the control and the yeast groups. Spatiotemporal maps were created from video recordings of baseline in the control group and the experimental group. The colon as well as the in‐ and outflow tubes are filled with phosphate buffered solution, and the fluid column in the outflow tube determines an intraluminal pressure of 4 cm H_2_O in the experiments described in this and all the other figures. (A) The baseline in the control group shows typical LDCs occurring over a long distance, having a fast speed of propagation and a contraction (black) and a relaxation (white) phase. (B–D) The baseline in the experimental group. After yeast extract gavage, the typical LDC became slower and remained contracted in the proximal colon for much longer. In (C), a proximal sustained contraction lasted for 790 s and propagated for 6.18 cm; in (D), strong RPMC activity is seen in the distal colon. This figure showed two slow‐propagating contractions with a lot of RPMCs in the distal colon.

Rats that received yeast extract generated LDCs that propagated over a more considerable length along the colon, from 8.38 ± 1.83 to 9.86 ± 1.95 cm (*p* < 0.05). Their duration increased markedly from 34.1 ± 10.44 to 66.15 ± 23.63 s (*p* < 0.05), while their amplitude, which was calculated by its black intensity value in the spatiotemporal map (the higher the value, the higher the amplitude), did not change significantly (*p* > 0.05). The LDC frequency and propagation velocity were inhibited compared to the control group: the frequency decreased from 0.41 ± 0.17 to 0.29 ± 0.10/min (*p* < 0.05), and the propagation velocity decreased from 0.35 ± 0.09 to 0.18 ± 0.08 cm/s (*p* < 0.01) (Table 1) (Figure 2A,B).

**TABLE 1:** Effects of yeast extract on LDCs.

LDCs	Frequency (/min)	Propagation length (cm)	Contraction duration (s)	Velocity (cm/s)	Black intensity
Yeast extract (*n* = 18)	0.29 ± 0.10*	9.86 ± 1.95*	66.15 ± 23.63*	0.1 ± 0.08**	51.58 ± 10.58
Control (*n* = 10)	0.41 ± 0.17	8.38 ± 1.83	34.1 ± 10.44	0.35 ± 0.09	46.45 ± 29.48
*p* Value	0.024	0.043	0.012	0.003	0.572

*Note*: Mean values ± SD *p < 0.05, **p < 0.01.

Abbreviation: LDCs, long‐distance contractions.

Yeast extract also changed the shape of the LDCs. In the yeast extract group, the typical LDCs showed two different forms (Figure 2C,D). One started as a proximal sustained contraction, then propagated to the mid colon. The mean contraction length was 8.00 ± 2.48 cm, and the contraction would persist for a long time, 330.9 ± 151.47 s, making the proximal colon stay contracted while the distal stayed relaxed with the presence of several RPMCs. The propagation velocity was 0.029 ± 0.01 cm/s (Figure 2C). The other form was a slow‐propagating ring contraction. It originated from the proximal and contracted slowly to the mid or the distal colon, lasting for a long time. Its mean propagation length was 7.75 cm, the contraction time was 420 s, with a velocity of 0.02 cm/s (Figure 2D).

### 3.2 Investigating the neurogenic component with TTX

**FIGURE 3:**
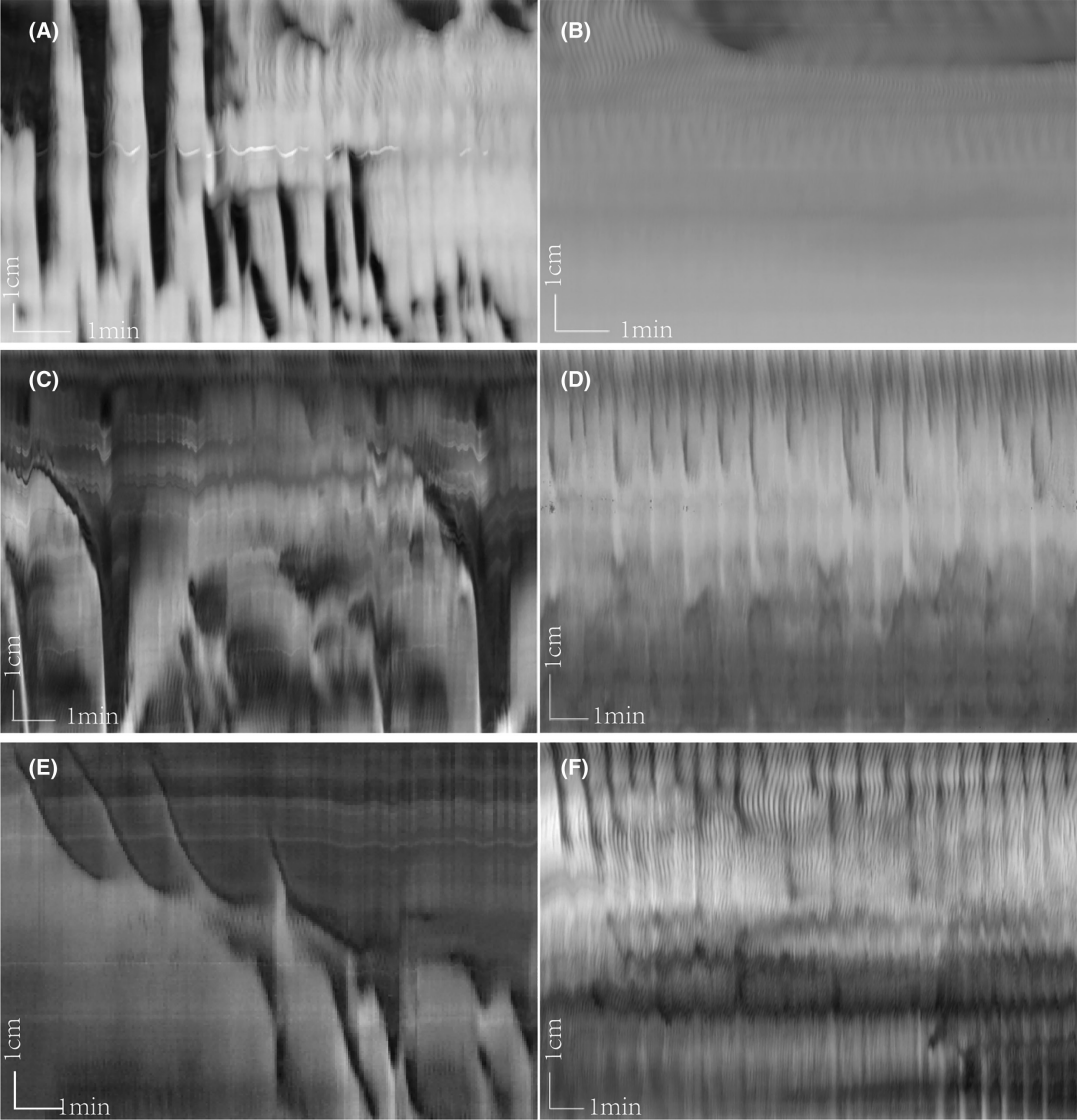
Tetrodotoxin (TTX) abolished long‐distance contraction (LDC) and rhythmic propulsive motor complex (RPMC) activity in both groups. (A) In the control group, spontaneous LDCs in the proximal and RPMCs in the mid and distal colon were prominent under baseline conditions. (B) TTX 2 μM (given 5 min before the start of the panel) abolished RPMC and LDC activity, only proximal ripples remained. (C, E) in the yeast group, the typical yeast‐induced LDCs occurred with its long‐lasting contraction in the proximal colon. (D, F) TTX 2 μM (given 5 min before start of panels) abolished LDC activity, but ripples and proximal strong rhythmic RPMCs remained at a higher frequency and propagating over a longer distance.

In the control group, TTX (0.2 μM) inhibited most motor patterns, including LDCs and RPMCs; only proximal ripples remained (Figure 3A,B). This indicates that distention‐induced activity is mediated by the ENS, except for ripples, which are purely myogenic. In the yeast extract group, the typical LDCs were abolished, and ripples remained. However, in contrast to the control group, rhythmic propulsive motor complexes (RPMCs) developed in the proximal and distal colon (Figure 3C–F). The frequency of RPMCs increased greatly from 0.95 ± 0.36 to 1.71 ± 0.40/min (*p* < 0.01), the RPMCs in the yeast group propagating further compared to the control group from 2.94 ± 1.04 to 4.43 ± 0.83 cm (*p* < 0.01), whereas the contraction duration decreased from 19.23 ± 8.83 to 12.81 ± 5.12 s (*p* < 0.05), with a faster velocity from 0.041 ± 0.018 to 0.067 ± 0.021 cm/s (*p* < 0.05) (Table 2).

**TABLE 2:** Effect of yeast extract on RPMCs after TTX.

	Frequency (/min)	Propagation length (cm)	Contraction duration (s)	Velocity (cm/s)
Yeast extract (*n* = 18)	1.71 ± 0.40**	4.43 ± 0.83**	12.81 ± 5.12*	0.067 ± 0.021*
Control (*n* = 10)	0.95 ± 0.36	2.94 ± 1.04	19.23 ± 8.83	0.041 ± 0.018
*p* Value	<0.001	<0.001	0.026	0.04

*Note*: Mean values ± SD *p < 0.05, **p < 0.001.

Abbreviations: RPMCs, rhythmic propulsive motor complexes; TTX, tetrodotoxin.

### 3.3 The myogenic component, observed in the presence of TTX

**FIGURE 4:**
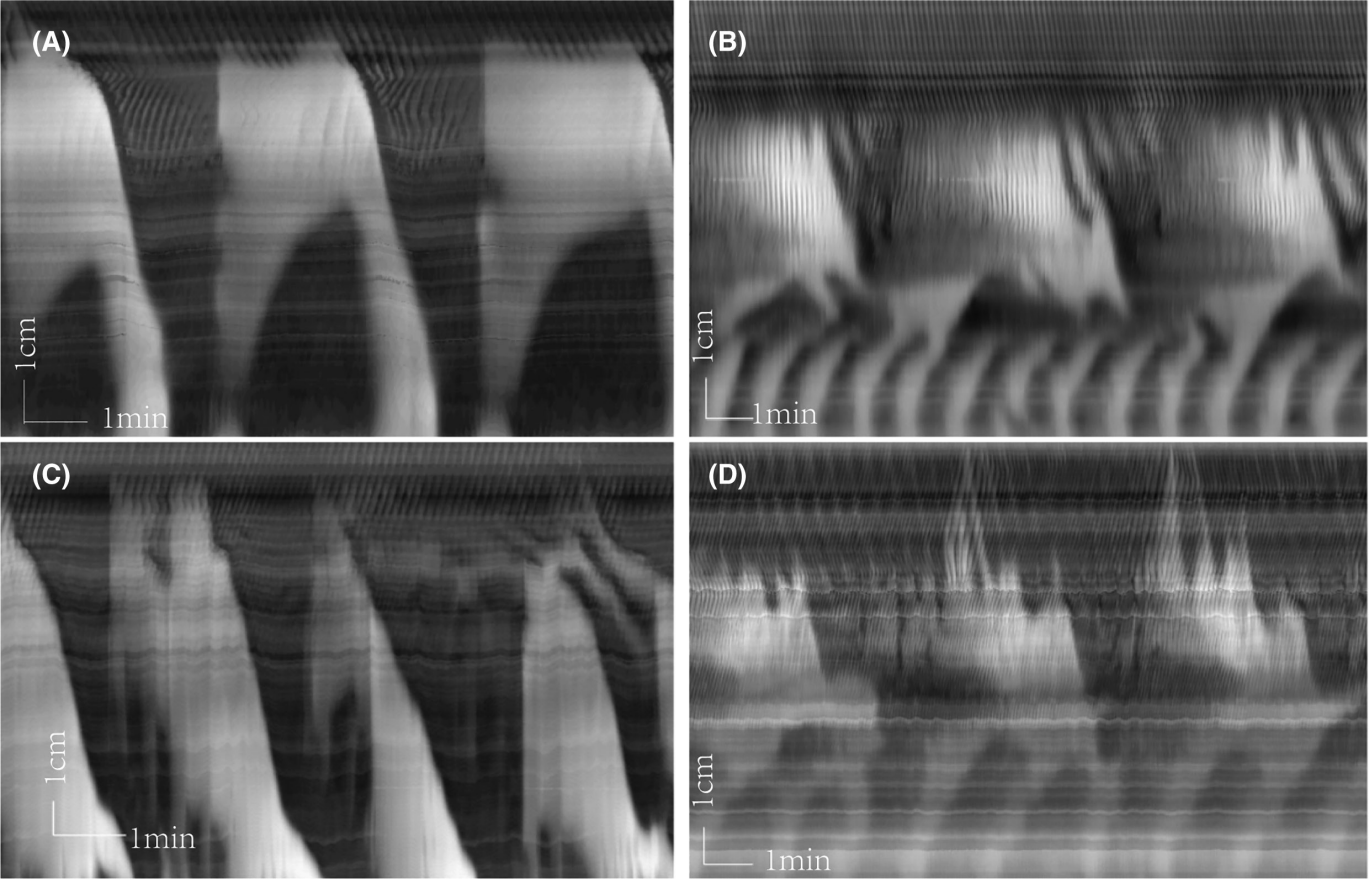
Effect of yeast extract on retrograde contraction. (A, C) in the control group, in the presence of bethanechol after tetrodotoxin, strong LDC‐like activity developed, with distal retrograde contractions. (B, D) in the yeast extract group, in response to bethanechol, the frequency and velocity of rhythmic retrograde contractions in the distal colon increased markedly.

As shown previously, bethanechol, in the presence of TTX, evokes the typical LDC, indicating their intrinsic myogenic nature, and in addition, strong retrograde contractions appear in the distal colon.^24^ Also in the present study, in the control group, bethanechol (2 μM) showed strong LDC activity (Figure 4A–C) and retrograde contractions at the distal end of the preparation in between LDCs (Figure 4A). The most dramatic effect of yeast was the marked increase in the frequency of the distal retrograde contractions (Figure 4B–D) (Table 3). In addition, the typical yeast‐type LDC patterns with superimposed RPMC activity were present.

**TABLE 3:** Effects on distal retrograde contractions in the presence of TTX and bethanechol.

Distal retrograde contractio*n*	Frequency (/min)	Propagation length (cm)	Contraction duration (s)	Velocity (cm/s)
Yeast extract (*n* = 18)	0.54 ± 0.21**	4.66 ± 1.78	80.31 ± 15.74	0.067 ± 0.021*
Control (*n* = 10)	0.16 ± 0.06	4.74 ± 1.26	134.97 ± 72.13	0.041 ± 0.018
*p* Value	0.006	0.92	0.094	0.04

*Note*: Mean values ± SD *p < 0.05, **p < 0.01.

Abbreviation: TTX, tetrodotoxin.

## 4 DISCUSSION

Ingestion of yeast extract affected colonic motility. Yeast changed the characteristics of distention‐induced LDCs, the main propulsive motor pattern of the colon, making it stronger at a lower frequency. Proximal RMPCs were strongly present after TTX, indicating stronger myogenic activity. In addition, distal retrograde contraction increased greatly in the presence of TTX and bethanechol, again showing strong myogenic activity. Hence, yeast extract was excitatory to both myogenic and neurogenic activities.

### 4.1 Fermentation activity on the colonic motility

The human gut is densely populated with a diverse microbial community^33^, ^34^ including 1.5 kg of bacteria in the colon^35^ and a density of 10^12^ cells per gram of intestinal content.^36^ The gut microbiota closely interacts with the host through a broad array of molecular interactions affecting nutrition, immunity and metabolism throughout the lifespan.^37^ Yeast extract, as a fermented food, will interact with the microbes in the colon, producing metabolites such as acids, alcohols and carbon dioxide as major end products. In addition, a whole range of secondary metabolites, including vitamins, polyols or antioxidants, may bring specific health benefits.^14^ The most important end products of intestinal bacterial fermentation of dietary fibre reaching the colon are likely the short‐chain fatty acids (SCFAs), such as acetate, propionate and butyrate, which have beneficial effects on the host energy metabolism and inflammatory responses, as there are SCFA receptors and target molecules expressed in metabolic and immune tissues.^38^, ^39^ Gut microbiota, acting through SCFAs on enterochromaffin (EC) cells, promotes serotonin (5‐HT) production.^40^, ^41^ Our previous experiments indicated that 5‐HT was involved in the generation of colonic migrating motor complexes (LDCs and RPMCs) in the rat colon through 5‐HT_3_ and 5‐HT_4_ receptors.^32^ In addition, the acidification of colonic contents stimulates colonic contractions in guinea pigs.^42^ Yeast extract is obtained from natural yeast through autolyzing baker's yeast or brewers' yeast after fermentation and maturation; when the yeast extract acts with the microbes, it decreases the pH of the colon.^14^ Thirdly, gas produced from yeast fermentation may also promote colonic motility, as colonic distension resulting from gas production during fermentation could stimulate high‐amplitude propagated contractions in the human colon.^25^, ^43^ Hence, the effect of yeast extract on the activity of the colon may be related to the fermentation process in the colon.

### 4.2 The neurogenic and myogenic control of the colon with yeast extract

Colonic motility is mainly controlled by the intrinsic (enteric) and extrinsic autonomic nervous systems and the pacemaker activities generated by the interstitial cells of Cajal (ICC).

In in vitro studies, neural control will be confined to the ENS. The ENS is an integrated neuronal network localized along the gut and organized in two major plexuses: the myenteric and the submucosal plexuses. The myenteric neurons are mainly involved in the control of gastrointestinal motility.^13^, ^44^ The fact that LDCs and RPMCs are abolished by TTX in both groups indicates that under baseline distended conditions, the presence of the major motor patterns depends on neural excitation. In the yeast extract group, the LDCs propagated slowly, and their appearance mimicked the LDC‐like pattern obtained by adding TTX and bethanechol, suggesting that the yeast extract promoted activity through myogenic mechanisms.

Interstitial cells of Cajal are distributed throughout the gastrointestinal tract and are often abnormal in diseased states.^31^, ^45^ The networks of ICC are key players in the control of gastrointestinal motility for generating electrical pacemaker activity (rhythmic slow oscillations of membrane potential) that provide the musculature with the mechanism to produce propulsive rhythmic contractile activity,^31^, ^45^, ^46^ and are conduits for muscle innervation.^47^ The slow waves generated from the ICC associated with the submuscular plexus (ICC‐SMP) are the basis of ripple activity, while the ICC associated with the myenteric plexus (ICC‐MP) are involved in the generation of LDC and RPMC in cooperation with the ENS. Here, we confirm that ripples exist in the presence of TTX, whose frequency is the same as the slow wave generated by the ICC‐SMP, indicating their myogenic origin. In addition, the muscarinic agonist bethanechol evoked LDC‐like contraction in the presence of TTX in both groups, indicating that myogenic control guides the rhythmic initiation and propagation of propulsive motor patterns. In the yeast extract group, rhythmic RPMCs developed in the middle and distal colon after adding TTX, and typical LDCs were transformed into slow‐propagating LDCs with high intensity, just like the rhythmic contraction after TTX plus bethanechol, which strongly suggests that yeast extract enhances myogenic activity controlled by ICC and the musculature. Furthermore, after adding bethanechol, retrograde contractions showed a higher frequency and faster speed in the yeast extract group compared with the control group, again showing the excitation of myogenic contractions. As a food additive, yeast extract may function as a biotherapeutic for chronic constipation by promoting colonic motility by activating myogenic control mechanisms. Further research may reveal which pathways are involved in the effects of yeast extract, including interactions between ICC and serotonergic, cholinergic and nitrergic pathways.

### 4.3 Clinical relevance

A study on patients with severe delayed transit leading to chronic constipation showed that their microbiota were significantly altered; importantly, treatment with bisacodyl resulted in normalization of the faecal flora.^48^ Bisacodyl generates extensive HAPW activity in the human colon, the equivalent of the LDCs in the present study.^25^, ^49^ Hence, the first line of treatment for colonic microbial dysregulation may be the promotion of colonic motility. The significance of our present study is that yeast increases colonic motility, and that may be the major therapeutic effect on constipation, in addition to the restoration of a healthy microbiota, which includes the beneficial effect of producing butyrate.^50^ It appears that changes in microbiota are secondary to constipation: an abnormal microbiota may be caused by constipation. This is consistent with some studies that do not find abnormal microbiota in patients with chronic constipation.^51^ With respect to mechanisms of action, several studies do not find abnormal motor activities of the colon in patients with severe chronic constipation after they are studied in vitro when removed by surgery.^51^, ^52^ This suggests that colonic dysmotility leading to constipation may be primarily related to abnormal extrinsic autonomic control. It may therefore be that yeast products may interact with enteric or extrinsic autonomic nerves to increase colonic motility.^53^ Indeed, butyrate activates colonic motility via actions on the nervous system in rats^13^ and *Lactobacillus reuteri* was shown to enhance the excitability of colonic enteric neurons.^54^

## AUTHOR CONTRIBUTIONS

**Hongfei Li:** Conceptualization (lead); data curation (lead); formal analysis (lead); investigation (lead); methodology (lead); project administration (lead); writing – original draft (lead). **Yanzhao Ji:** Formal analysis (supporting); methodology (supporting); writing – review and editing (supporting). **Hesheng Luo:** Project administration (supporting); resources (lead); writing – review and editing (equal). **Jan D. Huizinga:** Conceptualization (supporting); methodology (supporting); supervision (equal); writing – review and editing (equal). **Ji‐Hong Chen:** Conceptualization (supporting); formal analysis (supporting); methodology (supporting); supervision (supporting); writing – review and editing (supporting).

## CONFLICT OF INTEREST STATEMENT

The authors have no conflicts of interests of any kind.

## Data Availability

All data are expressed in the manuscript. For additional information please contact the authors.
